# Identification of Novel Susceptible Genes of Gastric Cancer Based on Integrated Omics Data

**DOI:** 10.3389/fcell.2021.712020

**Published:** 2021-07-20

**Authors:** Huang Yaoxing, Yu Danchun, Sun Xiaojuan, Jiang Shuman, Yan Qingqing, Jia Lin

**Affiliations:** Department of Gastroenterology, Guangzhou First People’s Hospital, School of Medicine, South China University of Technology, Guangzhou, China

**Keywords:** gastric cancer, GWAS, integrated omics data, a machine learning based method, biomarkers

## Abstract

Gastric cancer (GC) is one of the most common causes of cancer-related deaths in the world. This cancer has been regarded as a biological and genetically heterogeneous disease with a poorly understood carcinogenesis at the molecular level. Thousands of biomarkers and susceptible loci have been explored via experimental and computational methods, but their effects on disease outcome are still unknown. Genome-wide association studies (GWAS) have identified multiple susceptible loci for GC, but due to the linkage disequilibrium (LD), single-nucleotide polymorphisms (SNPs) may fall within the non-coding region and exert their biological function by modulating the gene expression level. In this study, we collected 1,091 cases and 410,350 controls from the GWAS catalog database. Integrating with gene expression level data obtained from stomach tissue, we conducted a machine learning-based method to predict GC-susceptible genes. As a result, we identified 787 novel susceptible genes related to GC, which will provide new insight into the genetic and biological basis for the mechanism and pathology of GC development.

## Introduction

Gastric cancer (GC) is one of the most common malignant dangerous neoplasms and fatal diseases in the world. It has been reported that there were approximately one million newly diagnosed gastric carcinoma cases, which caused 780,000 deaths in 2018 (Bray et al., 2018). It is notable that nearly half of the GC incidences occurred in the Asian region, which partly resulted from the diverse hereditary background, behavioral factors, and the spread of and infection by *Helicobacter pylori* (Chen et al., 2016; Zheng et al., 2017). A lot of work has been done to improve the diagnosis and therapy of GC. However, the survival rate of GC patients remains poor at approximately 30% in the recent 5 years (DeSantis et al., 2014). Therefore, many efforts have been made to discover new biomarkers to help in staging and in prognosis of the tumor diagnosis, which could help in improving early diagnosis and prognostic prediction of GC (Ludwig and Weinstein, 2005).

Disease gene prediction is a task of identifying the significant susceptible genes related to diseases. There have been a variety of approaches proposed, such as annotation-based approaches, network-based approaches, and machine learning-based approaches. Annotation-based approaches, such as prioritization of candidate genes using statistics (POCUS) (Turner et al., 2003), SUSPECTS (Adie et al., 2006), Endeavor (Aerts et al., 2006), and Transcriptome Ontology Pathway PubMed based prioritization of Genes (ToppGene) (Chen et al., 2007), are proposed based on annotating the genes with respect to biological structures or functions then comparing the annotations with known disease causal genes. However, these methods have a limitation of failing to capture the indirect relationships between the genes that may have common features or functions but are still not annotated. Network-based methods are proposed to overcome this by utilizing the large scale of interactome data between cellular molecules covering most of the genome and proteome (Wang et al., 2011).

Machine learning techniques have been applied to solve various biomedical problems (Lu and Zhao, 2019; Zhao et al., 2020a, b), such as pattern recognition (Barral et al., 2012; Cui et al., 2020), classification (Basford et al., 2013; Zhao et al., 2021a), prediction of drug target (Ding et al., 2014; Tianyi et al., 2020), and genome annotation (Yip et al., 2013). Thus, there is no doubt that machine learning methods have been applied in disease-associated genes prediction (Calvo et al., 2006; Xu and Li, 2006). In recent years, emerging evidences have illustrated the essential role of single-nucleotide polymorphisms (SNPs) in GC development and progression. Since genome-wide association studies (GWAS) are a widely known powerful approach to explore complex susceptible variants of diseases, many studies have reported a number of susceptibility loci associated with GC through GWAS analysis; however, they can only explain a small fraction of GC heritability (Wang et al., 2017; Park et al., 2019). Moreover, most disease-related SNPs identified by GWAS fall into intergenic or non-coding regions, which may influence the process of pathogenesis by modulating the expression level of target genes (Maurano et al., 2012). However, genetic variants are still powerful and high-quality biomarkers for screening GC susceptibility (Mocellin et al., 2015).

In addition, it has been proven that gene expression is significantly related to diseases (Zhao et al., 2021b). Expression quantitative trait locus (eQTL) analysis has been regarded as a powerful approach to provide prior weights for the statistical analysis of new causal SNP identification and prioritize SNPs or genes for further validation (Li et al., 2013b). Due to the theory of linkage disequilibrium (LD), which is reflected by the non-random association of alleles of different loci, it can be inferred that SNPs can regulate the pathologies of diseases by modulating the expression level of target genes. However, most studies select the representative SNPs by their closest located gene, which may inevitably obscure the genetic effect between that candidate gene and the trait. Thus, integrating GWAS data and eQTL data can help us to detect the genetic mechanism of complex disease. The Genotype-Tissue Expression (GTEx) project has provided the largest comprehensive public database of whole-genome and transcriptome sequencing data to help better understand the effects and molecular mechanism of function variations.

Considering the fact that regulatory causal SNPs may exert their function by affecting their target gene expression, we collected 1,091 GC cases and 410,350 controls from GWAS catalog database and eQTL summary data from stomach tissues in the GTEx database (Rashkin et al., 2020). We then extracted gene features from both GWAS summary data and eQTL data and integrated them as a 10D vector to represent gene feature, then we performed several machine learning methods to assess the classification performance of the models and selected random forest (RF) classifier based on its excellent performance. We identified 787 novel susceptible genes related to GC which may help provide new insights into the mechanism of GC.

## Materials and Methods

### GC GWAS Datasets

In this study, we obtained a GC GWAS dataset consisting of 1,091 cases and 410,350 controls from the UK Biobank (Rashkin et al., 2020). All the subjects were genotyped with Affymetrix Genome-Wide Human SNP Array. Eleven significant susceptible SNPs were identified related to GC and esophageal carcinoma with *p*-value < 1×10^−6^. However, they identified 9,986,610 susceptible loci associated with stomach and esophageal cancer in total, which is utilized in our study to be further identified.

### Tissue eQTL Dataset

Expression level-associated SNPs in stomach tissues were obtained from the GTEx v8 database. Genotyping was performed utilizing Illumina HumanOmni 5 and 2.5 M. And transcriptome dataset was generated by using Affymetrix Expression Array or Illumina TruSeq RNA sequencing. As a result, 24,291 susceptible loci were identified based on gene expression level.

### GC-Related Genes and Candidate Genes

After obtaining both omics summary data, we obtained 5,632 gastric-related genes from DisGeNET database, which are considered as positive genes for machine learning methods (Bauer-Mehren et al., 2010). Then we downloaded the human gene networks from HumanNet v2.0, which was a database of human gene networks illustrating gene–gene interactions (Hwang et al., 2018). After filtering the genes related to the GC causal genes obtained from DisGeNET, we obtained 3,227 genes whose correlation scores were <1, which indicate that they are negative genes, and the rest of the genes are regarded candidate genes which may have association with GC.

### Feature Extraction

We first obtained GWAS dataset and eQTL dataset associated with GC from the GWAS catalog database and the GTEx database, which represent the gene feature from the aspect of phenotype and transcription, respectively. Since the LD correction structure means that the majority of the identified variants associated with the traits frequently point to the regions where many genes are located, it is extremely difficult to prioritize among these susceptible genes to identify the most functionally relevant causal genes merely based on GWAS data. It is widely known that SNPs can exert their regulation function by modulating the expression level of target genes, which may further have a significant influence on the phenotype. Many studies have applied analytical approaches to integrate eQTL and GWAS data to detect the causal genes associations with complex traits (Giambartolomei et al., 2014; Gusev et al., 2016). However, to our knowledge, there is no method utilizing machine learning classifiers to prioritize susceptible genes of GC based on integrated GWAS and eQTL summary data.

After obtaining both omics summary data, we obtained gastric-related genes from DisGeNET database (Piñero et al., 2016). We first performed a data preprocessing process to transform the names of genes downloaded from different databases. We used an R package to obtain the detailed information of the genes, such as chromosome, start position, and end position information of these genes. After mapping these genes to the GWAS and eQTL data based on the location information, we finally got 5,633 genes regarded as a training set. According to the mapping information, we kept the genes with at least one susceptible loci identified by GWAS analysis. After prioritizing the SNPs by their *p*-value obtained by GWAS analysis, we used the *p*-value of the top five SNPs related to each gene as a 5D phenotype-based feature vector. For those genes with less than five associated SNPs, the feature vector is filtered with 1, which means the gene has no correlation with GC. Thus, the phenotype-based feature vector of *G_i_* can be denoted as follows:

(1) $$G_{i}^{p}=\left[\begin{array}{ccc} P_{p}^{1}, & \begin{array}{cc} P_{p}^{2}, & P_{p}^{3}, \end{array} & \begin{array}{cc} P_{p}^{4}, & P_{p}^{5} \end{array} \end{array}\right]$$

After obtaining the top five SNPs associated with these genes, these SNPs can be mapped to the stomach tissue eQTL data. Based on the same method, we use the *p*-value of those SNPs successfully mapped in eQTL data to represent a 5D transcriptome-based feature vector of each gene. For those SNPs not mapped, we all use 1 to fill up the feature vector. Thus, the transcriptome-based feature vector can be denoted as follows:

(2) $$G_{i}^{T}=\left[\begin{array}{ccc} P_{T}^{1}, & \begin{array}{cc} P_{T}^{2}, & P_{T}^{3}, \end{array} & \begin{array}{cc} P_{T}^{4}, & P_{T}^{5} \end{array} \end{array}\right]$$

Thus, each gene feature can be represented as a 10D feature vector based on the integrated omics data.

### Gene Prediction Using Random Forest Classifier

**FIGURE 1:**
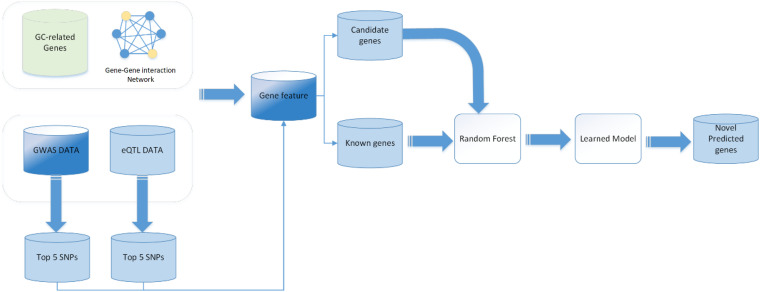
Workflow of gastric cancer-related genes prediction.

To date, the binary classification methods have been widely applied in disease causal gene prediction problems, such as naïve Bayesian classifier (NB), support vector machine (SVM), RF, and some deep learning methods such as convolutional neural network (CNN), graph neural network (GCN), and deep neural network (DNN). In this work, we used a RF classifier to predict GC causal genes. In order to evaluate the RF classifier, we performed a 10-fold cross-validation on the training set. The main workflow is shown in Figure 1. First, the 5,633 genes are randomly divided into 10 groups; 9 of them were chosen to be training samples, and the last one is the testing set for a total of 10 training iterations. Grid searches were performed to obtain the best performance of the parameters of the RF classifier. The final statistical results are averaged after 10 iterations. The receiver operating characteristic (ROC) curve and the area under the curve (AUC) are utilized to assess the performance of the classifier. The ROC curve is created by plotting the true positive rate (TPR) against the false positive rate (FPR) at various threshold settings to illustrate the diagnostic ability of a binary classifier system. This measure is related to the evaluation criteria TPR, which can be denoted as follows:

(3) $$\text{TPR}=\frac{TP}{TP+FN}$$

where *TP* means true positive conditions, and *FN* means the false negative conditions. TPR is also known as sensitivity or recall. Another measurement is true negative rate (TNR), which is also known as specificity or selectivity; TNR can be denoted as follows:

(4) $$\text{TNR}=\frac{TN}{TN+FP}$$

Another measurement is FPR, which can be denoted as follows:

(5) $$\text{FPR}=\frac{FP}{FP+TN}=1-\text{TNR}$$

where *FP* indicates the false positive results and *TN* indicates the true negative results. AUC means the AUC. The AUC of each iteration is shown in Table 1. After the model is trained, we use the model to predict the candidate genes.

**TABLE 1:** Area under the curve of 10-fold cross-validation.

	AUC
1	0.881
2	0.91
3	0.894
4	0.886
5	0.878
6	0.872
7	0.896
8	0.892
9	0.902
10	0.909
Average	0.892

## Results

### Performance Comparison Over All Known Disease Genes

**FIGURE 2:**
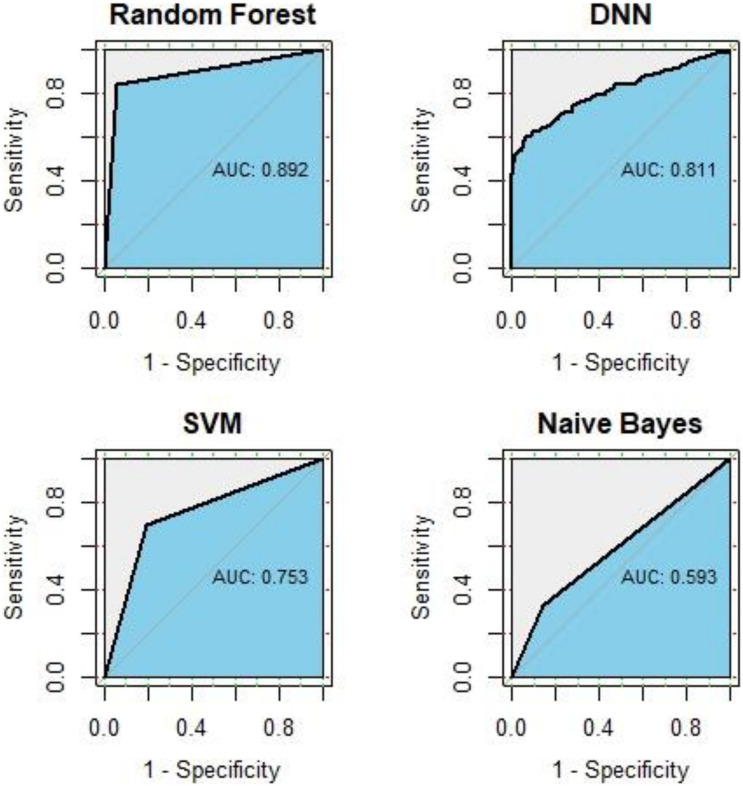
Comparison of prediction performance.

In this study, we use all known GC-related genes obtained from DisGeNET as positive training samples, and genes with correlation scores under 1 were obtained from HumanNet v2.0 as negative training samples. We compared the predictive performance of RF, SVM, NB, and DNN. After 10-fold cross-validation of each method, the ROC curve and average AUC value is shown in Figure 2. The AUC of RF is 0.892, followed by the AUC of 0.811 of DNN, an AUC of 0.753 of SVM, and an AUC of 0.593 of NB, which means RF did the best performance in disease gene classification. For details, the AUC value of each iteration in RF classifier is shown in Table 1.

### Case Study

We obtained 7,406 candidate genes from HumanNet v2.0, and then extracted the feature representation of each gene. Then we used the trained RF classifier to predict the prioritizing genes associated with GC. The top 24 susceptible genes are shown in Table 2. From Table 2, 11 of the 24 predicted genes are reported to have direct or indirect association with GC. For example, CD38 has been determined to be expressed at higher levels in the IL-10-producing Breg cells of GC patients (Wang et al., 2015). The study of Cheng et al. found that compared to control tissues, RARB messenger RNAs were significantly reduced in human gastric tumor samples (Cheng et al., 2013). As an indirect evidence, Wen et al. found that NRAS can be a target gene of miR-26a to improve the sensitivity of GC cells to cisplatin-based chemotherapies, which can be an evidence for the potential function of NRAS in chemotherapy for GC (Wen et al., 2015). GIPR, short for gastric inhibitory polypeptide receptor, has been regarded as a promising target for imaging and therapy in gastric and neuroendocrine tumors, and it has also been reported that GIPR is significantly overexpressed in stomach tissue compared with normal tissue (Sherman et al., 2013, 2014). In recent years, many studies have shown that PLAUR can be an effective prognostic biomarker and potential therapeutic target for GC due to the fact that the suppression of PLAUR could sensitize cancer cell death by inducing DNA damages (Li et al., 2013a; Ai et al., 2020). ANLN is a conserved actin-binding protein that exerts its functions in cytoskeletal dynamics during cell division and may affect cancer progression through Wnt/B-catenin pathway in GC.

**TABLE 2:** Predicted susceptible genes by random forest.

Symbol	Ensembl ID	Symbol	Ensembl ID
ANLN	ENSG00000011426	CD38	ENSG00000004468
TYROBP	ENSG00000011600	PDK4	ENSG00000004799
MATR3	ENSG00000015479	RARB	ENSG00000077092
NCDN	ENSG00000020129	ITGA2B	ENSG00000005961
TYMP	ENSG00000025708	CRLF1	ENSG00000006016
ALG1	ENSG00000033011	NRAS	ENSG00000213281
KRAS	ENSG00000133703	GTF2IRD1	ENSG00000006704
CDH1	ENSG00000039068	CACNA2D2	ENSG00000007402
BEST2	ENSG00000039987	DNAJC11	ENSG00000007923
TNFRSF17	ENSG00000048462	RPS20	ENSG00000008988
ADAMTS6	ENSG00000049192	GIPR	ENSG00000010310
PLAUR	ENSG00000011422	SLC6A7	ENSG00000011083

## Conclusion

Gastric cancer is one of the most malignant neoplasms in human health around the world causing approximately 10% of all cancer deaths. The main therapeutic strategies of GC include two ways: surgery and chemotherapeutic regimens. Thus, it is important to identify the susceptible genes in order to better understand the pathologies of the disease, which can further help in drug designing. Machine learning methods have been used in predicting the functions of unclassified or unannotated genes by utilizing genomic features (Schläpfer et al., 2017). In this study, we combined machine learning methods with genetic association data (GWAS analysis) and gene expression data (eQTL) to advance our understanding of GC etiology and pathology. Considering the LD, genes located close to susceptible loci identified by GWAS analysis may not be the causal genes of the disease. Since SNPs may also influence the expression level of the gene, the genes with different genotypes of the genetic variant will show differences in phenotype, which means that SNPs can also show effects on the diseases or traits. Therefore, we performed a RF classier on the collected 1,091 cases and 410,350 controls from GWAS dataset, integrated with stomach tissue eQTL data, to identify genes whose expression levels were associated with GC due to its causality. Compared to three other widely used binary classifiers, SVM, NB, and DNN, RF has the best performance in classifying GC-related genes. Since the overall importance score from the RF classifier is a sum of multiple individual importance scores, and each individual importance score is obtained from an average over multiple trees and cross-validations, the gene importance score can be regarded as not being affected by LD.

It is widely known that the accuracy of the prediction of genetic risk of complex diseases varies greatly between different diseases due to the heritability, phenotype, and the power and the amount of reported variants. Though GC is the most common cancer and causes a high mortality rate around the world, most studies only focused on the prognosis and treatment of GC. In this study, we identified 787 novel susceptible genes related to GC and focused on the top 24 of the susceptible genes. We found that CD38, RARB, NRAS, GIPR, PLAUR, ANLN, etc. have strong association with GC and have been reported to be related with GC indirectly; for example, they impact other pathways or exert their function by cooperating with other genes. However, we identified 787 novel susceptible genes related to GC, which is helpful in further studies to understand the etiology and pathology of GC and may be a strong theoretical basis for drug designing.

We used a machine learning method to identify GC-related genes. Although we have proven the effectiveness of our method by cross-validation and accuracy of our results by case study, biological experiments are still necessary to further reveal pathogenic mechanisms.

## Data Availability Statement

The original contributions presented in the study are publicly available. This data can be found here: GWAS dataset: downloaded from GWAS Catalog, accession: GCST90011807.

## Ethics Statement

Ethical review and approval was not required for the study on human participants in accordance with the local legislation and institutional requirements. Written informed consent for participation was not required for this study in accordance with the national legislation and the institutional requirements. Written informed consent was not obtained from the individual(s) for the publication of any potentially identifiable images or data included in this article.

## Author Contributions

HY and JL designed the study. HY, YD, and SX interpreted the data. HY, JS, and YQ analyzed the results. HY, YD, and JL were major contributors in writing and revising the manuscript. All authors read and approved the final manuscript.

## Conflict of Interest

The authors declare that the research was conducted in the absence of any commercial or financial relationships that could be construed as a potential conflict of interest.
